# Exploring the Psychological Stress, Anxiety Factors, and Coping Mechanisms of Critical Care Unit Nurses During the COVID-19 Outbreak in Saudi Arabia

**DOI:** 10.3389/fpubh.2021.767517

**Published:** 2021-11-26

**Authors:** Shaimaa Ahmed Awad Ali, Samar Salah Eldin Mohamed Diab, Ehab Kotb Elmahallawy

**Affiliations:** ^1^Department of Nursing, College of Applied Medical Sciences, Jouf University, Sakaka, Saudi Arabia; ^2^Critical Care and Emergency Nursing Department, Faculty of Nursing, Mansoura University, Mansoura, Egypt; ^3^Nursing Department, Faculty of Nursing, Menoufia University, Menoufia, Egypt; ^4^Department of Zoonoses, Faculty of Veterinary Medicine, Sohag University, Sohag, Egypt

**Keywords:** COVID-19, psychological stress, anxiety factors, coping mechanisms, critical care nurses

## Abstract

**Background:** The spread of coronavirus disease 2019 (COVID-19) throughout the world leads to a series of modifications of several National Health Service organizations, with a potential series of psychological consequences among nurses.

**Methods:** This study was undertaken to assess the psychological stress, anxiety factors, and coping mechanisms of critical care unit nurses during the COVID-19 outbreak. A cross-sectional research design was employed, and the convenience sample consisted of 469 nurses working at several hospitals in Saudi Arabia during the period from July to September 2020. This study used the Generalized Anxiety Disorder, Coping Mechanism, and Nursing Stress scale.

**Results:** Interestingly, more than one-third and one-quarter of the studied nurses had severe and moderate anxiety levels, respectively. In addition, the most anxiety-causing factors included providing care for their infected colleagues and worrying about infecting their families. More than one-quarter and slightly less than half of the studied nurses had high and moderate stress levels, respectively. Furthermore, more than half of the participants had low coping mechanisms and one-quarter had moderate coping mechanisms. In addition, there was a strong positive correlation between anxiety and stress levels, and there was a strong negative correlation between coping mechanisms and stress and anxiety levels.

**Conclusions:** Collectively, this study explored the psychological stress, anxiety factors, and coping mechanisms among critical care unit nurses during the COVID-19 outbreak in Saudi Arabia. Continuous educational programs for nurses on using coping mechanisms should be developed in combination with teaching preventive measures for defining a psychological intervention plan within a mandatory occupational health surveillance program. This study recommends that constructive planning and necessary provision of supportive measures by the legal authorities and policymakers protect nurses and minimize their psychological stress to fulfill high-quality nursing care.

## Introduction

In December 2019, multiple unexplained cases of pneumonia were reported in Wuhan, Hubei province, China. Epidemiological findings revealed severe human-to-human transmission, which was later confirmed to be caused by a novel coronavirus infection. The WHO named it as coronavirus disease 2019 (COVID-19) (1). The COVID-19 crisis has been characterized as the biggest challenge for the world since World War II due to the resulting health crisis. Importantly, the COVID-19 pandemic results in a wide range of disruptive respiratory and digestive symptoms. These symptoms range from mild self-limiting symptoms to acute pneumonia, acute respiratory distress syndrome, septic shock, and even multiple failure syndromes of the body systems (2). Droplets and direct contact, among others, are considered as the major sources of infection by COVID-19. Importantly, the world faced a slowdown or even a complete shutdown of daily activities during the first two waves of this pandemic. Moreover, individuals were encouraged to implement social distancing to reduce the transmission of the infection (3). Taking this into consideration, there are no specific available drugs and vaccines for combating the infection during the first wave of this pandemic, and treatment relied on antiviral therapy, isolation, and symptomatic support in combination with a close monitoring of the progression of the disease (4, 5), then the last year witnessed the development of vaccines for combating the pandemic (4–9).

It is noteworthy to state that health-care professionals faced many challenges resulting from an exponential increase in the demand for healthcare during COVID-19. These challenges included long work shifts, few resources, precarious infrastructure, and the lack of sufficient protective clothing also, many health-care workers felt unprepared to conduct the clinical intervention of patients infected with a new virus with no established clinical protocols or treatments (10). Taking this into account, frontline health-care staff members are one of the most vulnerable groups because they constantly deal with the threat of COVID-19 infection (11). It should be stressed that COVID-19 has been considered an emerging and easily clustering infectious disease (3). Because of the highly infectious nature of and limited knowledge about COVID-19, health-care workers are under extreme physical and psychological pressure while on duty (12). They are not only at an elevated risk of becoming infected but also having been reported to experience related depression, anxiety, insomnia, physical discomfort, difficulty breathing, stigma, and frustration (13). On reviewing the available literature, several studies have shown that the group of health-care workers who are in direct contact with patients are exposed to the highest levels of risk for contracting COVID-19 (14, 15). Nurses are particularly vulnerable to many job-related hazards and undergo a considerable amount of emotional pressures in relation to their jobs because of their long, intense exposure to various stressors (16). Clearly, it is important to note the nature of the coping strategies used by these health-care and emergency workers in these situations and their effectiveness in terms of reduction and effectively coping up with stress. Indeed, the effective management of stress levels in the acute/emergency phase could reduce the risk of developing long-term stress or other pathologies, such as anxiety and depression (17). Importantly, providing social, moral, and psychological support services is urgently needed and should be based on coping strategies for managing stress mechanisms, which should go together with the provision of facilities and equipment by hospital managers and the government. The psychological intervention plan should include two pillars: (a) providing health-care workers with adequate information, training, and personal protective equipment to tackle the COVID-19 emergency and (b) enhancing the emotional skills of health-care workers to deal with anxiety by offering psychological support. Psychologists providing emotional support to patients and health-care personnel are also urgently needed (18). Given the aforementioned information, this study aimed to assess the psychological stress, anxiety factors, and coping mechanisms among critical care unit nurses during the COVID-19 outbreak by addressing the following research questions.

- Q1: What was the stress level among nurses during the COVID-19 outbreak in critical care units?
- Q2: What was the anxiety level among nurses during the COVID-19 outbreak in critical care units?
- Q3: What are the coping mechanisms of nurses during the COVID-19 outbreak in critical care units?
- Q4: What were the factors causing anxiety among nurses during the COVID-19 outbreak in critical care units?
- Q5: Is there a correlation between psychological stress and anxiety levels and coping mechanisms?

## Methods

### Ethical Approval

This study was conducted with the approval of the research ethics committee of Jouf University (Approval No. 05-06-42). The submission of answers to the questionnaire was considered by giving consent to take part in this study. Confidentiality of the study subjects' data was maintained throughout this study by making the data nameless.

### Research Design and Setting

A cross-sectional study was conducted from July to September 2020 at the Adult Intensive Care Units of several receiving hospitals (*n* = 6) in Saudi Arabia. The hospitals involved in this study were Arar Central Hospital, Arar; Qurayyat Public Hospital, Qurayyat; Prince Mohammed Ben Abdel-Aziz Hospital; Riyadh King Abdul Aziz Specialized Hospital; Prince Mutaib bin Hospital; and the Maternity and Children's Hospital pediatric care unit and neonatal intensive care units in Sakaka City, Jouf region, Saudi Arabia.

### Subjects and Instruments

The convenience sample included 469 nurses working at the abovementioned settings and were enthusiastic to participate in this study; 58.2% of them were women, 67.2% were married, 50.7% had a bachelor's degree in nursing, 41.8% of them were bedside nurses, and the mean age was 31.73 ± 5.6 years, and the mean years of experience was 8.91 ± 2.35 years. The following tools were used.

*Tool I*: Psychological responses of nurses toward caring for critically ill patients with COVID-19, which consisted of two parts.

*First part*: The Generalized Anxiety Disorder-7 item test (GAD-7) (19) was used. It consisted of seven items that measured worry and anxiety symptoms. Each item was scored on a 4-point Likert scale (0–3) with total scores ranging from 0 to 21 with higher scores reflecting greater anxiety. Scores above 10 were considered to be in the clinical range (19). GAD-7 has been shown to have good reliability and construct validity (20). These scores were summed and converted into a percent score. The results were classified into three categories: severe anxiety if the score was >70%, moderate anxiety if the score was 50–70%, and low anxiety if the score was <50%.

*Second part*: The second section investigated 12 factors that could induce anxiety in the nursing staff that were adapted from Tam et al. (21). Responses included the four choices ranging from 0 to 3 (0 = not at all; 1 = slightly; 2 = moderately; 3 = very much).

*Tool II*: The coping mechanisms of nurses regarding COVID-19 were adapted from another previous study (22). The test consisted of 11 questions that looked at different personal coping strategies that the staff could have used. It initially comprised a yes or no response. Those who answered yes then rated the strategies from 0 to 4 (0 = never; 1 = sometimes; 2 = often; 3 = always). These scores were summed and converted into a percent score. The results were classified into three categories: a high coping mechanism if the score was >70%, a moderate coping mechanism if the score was 50–70%, and a low coping mechanism if the score was <50%.

*Tool III*: The Nursing Stress scale was adopted from a previous study (23). The scale consisted of 34 items that were distributed into 7 heterogeneous and potentially stressful situations, including death and dying patients (7 items), conflict with physicians (5 items), inadequate preparation (3 items), lack of staff support (3 items), conflict with other nurses (5 items), workload (6 items), and uncertainty concerning treatment (5 items). A 4-point Likert scale was used to indicate the frequency of work stressors experienced by nurses ranging from never (1), occasionally (2), and frequently (3) to very frequently (4). These scores were summed and converted into a percent score. The results were classified into three categories: a high coping mechanism if the score was >70%, a moderate coping mechanism if the score was 50–70%, and a low coping mechanism if the score was <50%. We used an online survey and email, Facebook, WhatsApp, and telegram services to collect the data from the subjects to maintain the rules of social distancing and limit the spread of COVID-19. The Google form (https://docs.google.com/forms/d/e/1FAIpQLSf7YLpKyVieUF_QewKwIIJRiaFQZ0XJxa3pmpzKG38F4pkjQQ/viewform?usp=sf_link) permits questionnaire design, the collection of data, a descriptive analysis of results, and the download of data through excel spreadsheets for extra analysis.

### Pilot Study

The pilot study was conducted on 49 participants who represented 10.44% of the total sample at the abovementioned settings to test the applicability of the constructed tools and the clarity of the included tools. Additionally, this pilot study aimed to assess the reliability and validity of developing a tool before its use in this study. This pilot study also estimates the time needed for each subject to complete the questionnaire.

### Validity and Reliability

A group of five experts in the critical nursing departments ascertained the content's validity to assess the layout, format, accuracy, consistency, and relevancy of the tools. Reliability pretesting was conducted using Cronbach's α for GAD-7, and the result was 0.894, the stress scale value was 0.914, and the coping mechanism score was 0.855.

### Statistical Analysis

The data collected from the pilot sample were revised, coded, and entered into a personal computer. Computerized data entry and statistical analysis were fulfilled using the Statistical Package for Social Sciences version 24. Data were presented using descriptive statistics in the form of number and percent. Pearson's correlation coefficient analysis was used to measure the linear correlation between the two sets of data. Multiple linear regression (MLR), also known simply as multiple regression, was performed. This statistical technique used several explanatory variables to predict the outcome of a response variable.

## Results

The sociodemographic characteristics of the study participants are shown in Table 1 and divided into subgroups. As depicted, 58.2% of the sample participants were women, 67.2% were married, and 65.7% had children and were from Saudi Arabia. Approximately half of the sample (50.7 %) had a bachelor's degree. Moreover, 41.8 and 44.8% of the participants were bedside nurses and had 6–10 years of experience in an intensive care unit, respectively. Participants from different genders had a moderate score level of GAD-7 scale for fear, anxiety, stress, and coping. However, the male participants had a high mean score when compared to women (Table 2). Furthermore, as presented in Table 3, 35.8% of the participants felt nervous, anxious, and restless, and were very worried about different things as a result of COVID-19. In addition, 31.3% of the participants were not able to stop or control worrying. Moreover, 38.8% of the participants felt afraid as if something awful might happen and 34.3% of them had a trouble to be in a relaxed atmosphere. Also, 35.8% of the participants became easily annoyed or irritable for several days (Table 3).

**Table 1:** Characteristics of the study participants (*n* = 469).

**Characteristics of the study participants**		**No**.	**%**
Age (years), mean (SD)	31.73 (5.66)		
Gender	Male	196	41.8
	Female	273	58.2
Marital status	Married	315	67.2
	Not married	154	32.8
Have children	Yes	308	65.7
	No	161	34.3
Number of children	1–2 children	112	23.9
	3–4 children	126	26.9
	>4 children	70	14.9
	No children	161	34.3
Nationality	Saudi Arabian	308	65.7
	Not Saudi Arabian (Arabian)	98	20.9
	Not Saudi Arabian (Not Arabian)	63	13.4
Professional degree	Diploma	196	41.8
	Bachelor	238	50.7
	Postgraduate	35	7.5
Position	Matron	7	1.5
	Head nurse	119	25.4
	Supervisor	147	31.3
	Bedside nurse	196	41.8
Years of experience in the U	<1 year	42	9.0
	1–5 years	147	31.3
	6–10 years	210	44.8
	>10 years	70	14.9

**Table 2:** Level score of Generalized Anxiety Disorder toward coronavirus disease 2019 (COVID-19) according to critical care nurses' gender.

**Level of generalized anxiety disorder 7-item scale score**	**Male *N* (%)**	**Female*N* (%)**	**Total *N* (%)**	**MaleMean (SD)**	**Female Mean (SD)**	**Significance**
Minimal score (0–4)	0 (0)	49 (10.4)	49 (10.44)	0 (0)	2.71 (1.11)	0.00
Mild score (5–9)	56 (11.9)	56 (11.9)	112 (23.88)	7.63 (0.92)	7.75 (1.38)	0.85
Moderate score (10–14)	49 (10.4)	70 (14.9)	119 (25.37)	12.43 (1.81)	13 (1.15)	0.43
Severe score (15–21)	91 (19.4)	98 (20.9)	189 (40.29)	19.69 (1.97)	18.43(2.62)	0.72
Total	196 (41.79)	273(58.20)	469 (100)	13.96 (5.09)	12.0 (6.19)	0.17

**Table 3:** Distribution of the anxiety psychological responses of nurses toward caring for critically ill patients with COVID-19.

**Generalized anxiety disorder 7-item (GAD-7)**	**Not at all**	**Several days**	**More than half the days**	**Nearly every day**	**Mean (SD)**
	***N* (%)**	***N* (%)**	***N* (%)**	***N* (%)**	
1. Feeling nervous, anxious or on edge	35 (7.5)	147 (31.3)	119 (25.4)	168 (35.8)	1.90 (098)
2. Not being able to stop or control worrying	56 (11.9)	105 (22.4)	161 (34.3)	147 (31.3)	1.85 (1.0)
3. Worrying too much about different things	28 (6.0)	133 (28.4)	140 (29.3)	168 (35.8)	1.96 (0.94)
4. Trouble relaxing	21 (4.5)	154 (32.8)	161 (34.3)	133 (28.4)	1.87 (0.88)
5. Being so restless that it is hard to sit still	56 (11.9)	119 (25.4)	126 (26.9)	168 (35.8)	1.87 (1.00)
6. Becoming easily annoyed or irritable	42 (9.0)	168 (35.8)	147 (31.1)	112 (23.9)	1.70 (0.94)
7. Feeling afraid as if something awful might happen	63 (13.4)	119 (25.4)	182 (38.8)	105 (22.4)	1.70 (0.97)

**Figure 1:**
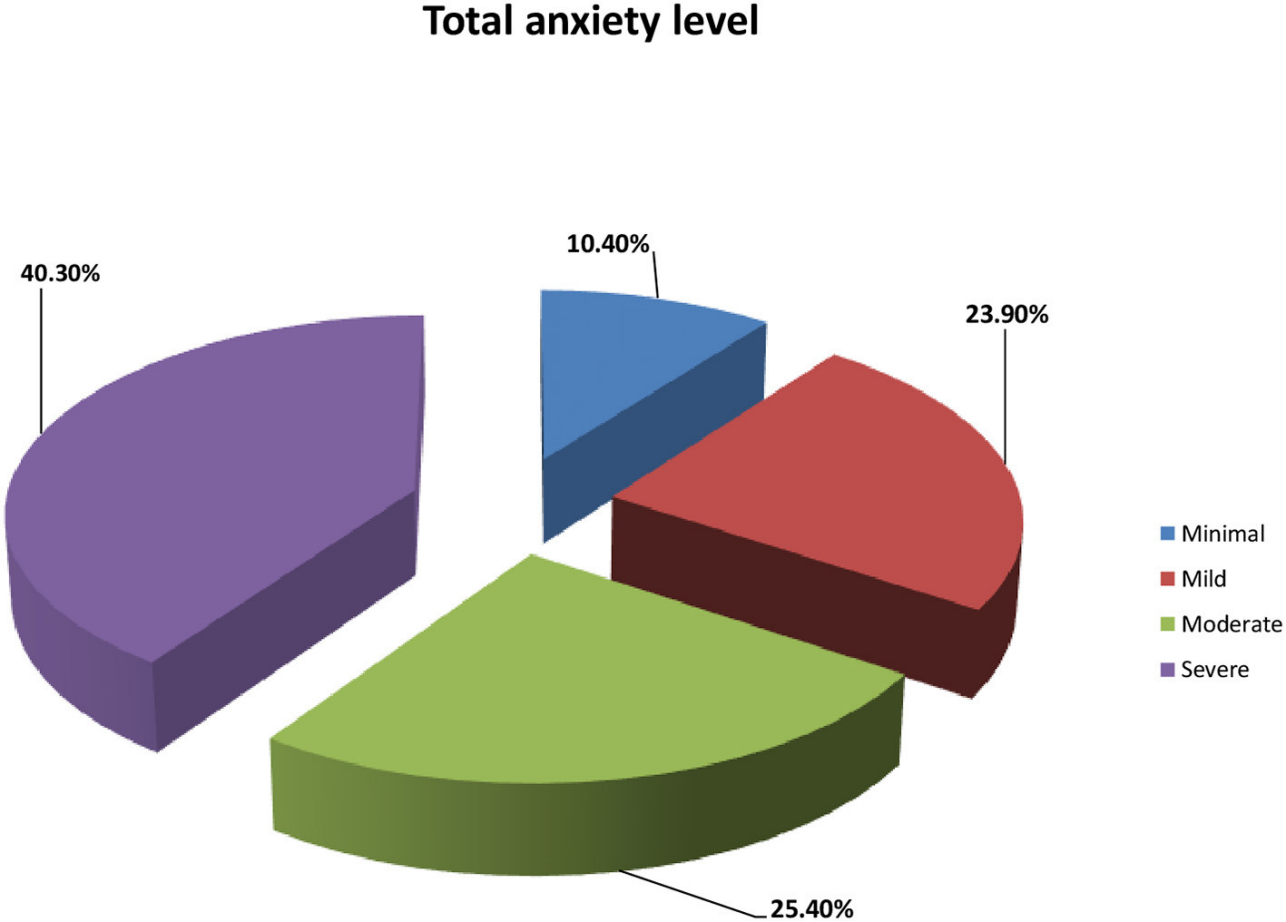
Distribution of the studied critical care nurses according to their total anxiety level (*n* = 469).

Table 4 shows that the distribution of the anxiety level scores and the factors “providing care for infected colleagues” and “worries about infecting their families” had the highest mean score [2.50 (0.58)], whereas thoughts that the current protection measures were still lacking, and constantly screening for infection had the lowest mean scores [1.04 (0.96) and 1.32 (0.67), respectively]. Conversely, 40.3 and 25.4% of the participants had severe and moderate anxiety levels, respectively (Figure 1). Additionally, 23.9 and 10.4% of the participants had mild and minimal anxiety, respectively (Figure 1). Importantly, Table 5 shows that “talking to yourself,” “motivating to face the COVID-19 outbreak with a positive attitude,” and “choosing solo transport modes,” such as self-driving and avoiding public transportation such as “subways” had the highest mean score [2.36 (0.67) and 2.34 (0.69), respectively]. “Venting emotions by crying” or “screaming and avoiding media news about COVID-19 and related fatalities” had the lowest mean score [1.04 (0.96) and 1.32 (0.67), respectively].

**Table 4:** Mean score of the studied nurses according to the factors that caused anxiety among the staff (*n* = 469).

**Factors that caused anxiety among the staff**	**Mean (SD)**
1. Seeing your colleagues were infected	2.39 (0.63)
2. You are worried about infecting your family	2.50 (0.58)
3. Small mistakes or inattentions can make you or others infected	2.46 (0.64)
4. Providing care for your infected colleagues	2.50 (0.58)
5. Seeing your infected patient die	2.43 (0.57)
6. New infections or suspected cases asking for your help	2.39 (0.63)
7. Lack of specific treatments for COVID-19	2.36 (0.68)
8. You were infected by an infected patient while working at the hospital	2.32 (0.67)
9. Seeing stress or fear from your colleagues	2.36 (0.68)
10. Constantly screening yourself for infection	1.32 (0.67)
11. Every day staying in protective clothing for a long time	2.29 (0.60)
12. You think the current protection measures are still lacking	1.04 (0.96)

**Table 5:** Distribution of the studied critical care nurses according to their coping mechanism (*n* = 469).

**Items**	**Never**	**Sometimes**	**Often**	**Always**	**Mean (SD)**
	***N* (%)**	***N* (%)**	***N* (%)**	***N* (%)**	
Following strict protective measures, such as hand washing and use of masks and protective clothing.	7 (1.5)	56 (11.9)	231 (49.3)	175 (37.3)	2.22 (0.71)
Every febrile patient may be infected with COVID-19, even if the nucleic acid test is negative.	14 (3.0)	35 (7.5)	280 (59.7)	140 (29.9)	2.16 (0.69)
Learning about COVID-19, its prevention, and mechanism of transmission.	0 (0)	56 (11.9)	224 (47.8)	189 (40.3)	2.28 (0.67)
Choosing solo transport modes, such as self-driving, and avoiding public transportation, such as subways.	0 (0)	56 (11.9)	196 (41.8)	217 (46.3)	2.34 (0.69)
Doing some leisure activities in your free time, such as watching movies and reading.	14 (3.0)	35 (7.5)	210 (44.8)	210 (44.8)	2.31 (0.74)
Chatting with family and friends to relieve stress and obtain support.	14 (30)	35 (7.5)	203 (43.3)	217 (46.3)	2.33 (0.75)
Talking to yourself and motivating yourself to face the COVID-19 outbreak with a positive attitude.	7 (1.5)	28 (6.0)	224 (47.8)	210 (44.8)	2.36 (0.67)
Seeking help from a psychologist.	42 (9.0)	28 (6.0)	231 (49.3)	168 (35.8)	2.12 (0.88)
Avoiding doing overtime to reduce exposure to patients with COVID-19 in the hospital.	21 (4.5)	35 (7.5)	259 (55.2)	154 (32.8)	2.16 (0.75)
Avoiding media news about COVID-19 and related fatalities.	63 (13.4)	112 (23.9)	189 (40.3)	105 (22.4)	1.72 (0.97)
Venting emotions by crying, screaming, etc.	245 (52.2)	98 (20.9)	77 (16.4)	49 (10.4)	0.85 (1.05)

**Figure 2:**
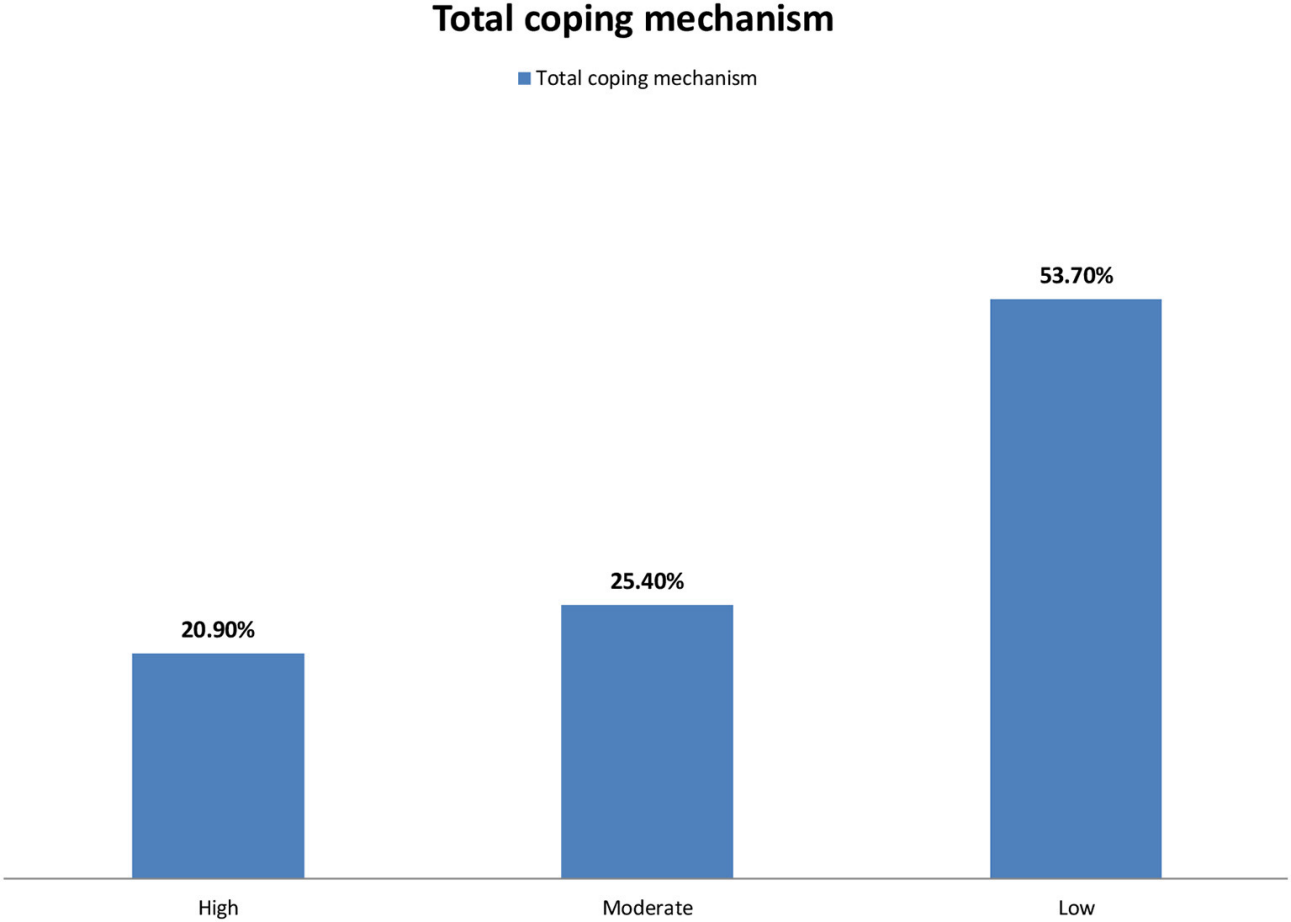
Distribution of the studied critical care nurses according to their total coping mechanism (*n* = 469).

In accordance with the total coping mechanism, as shown in Figure 2, 53.7% of the participants had low coping mechanisms, 25.4% of them had moderate coping mechanisms, and 20.9% had high coping mechanisms. Conversely, 41.8, 47.8, and 38.8% of the participants had high, moderate, and low levels of stress related to workloads, inadequate preparation, and conflicts with other nurses, respectively (Table 6). Furthermore, 29.8, 47.8, and 22.4% of the participants had high, moderate, and low levels of stress, respectively (Table 6). As shown in Table 7, there was a strong positive correlation between anxiety and stress levels (*p* < 0.01). Meanwhile, there was a strong negative correlation between coping mechanisms and stress and anxiety levels (*p* < 0.01). As shown in Table 8, a highly significant model was detected through the *F*-test value of 13.808 (*p* < 0.01). This model explained a 52% variation in the anxiety scale detected through the *R*^2^ value of 0.52. Also, the model explained that age and experience had a high-frequency negative effect on the level of anxiety (*p* < 0.01), while high education level had a slight negative effect on the level of anxiety (*p* < 0.05). In addition, bedside nurses had a high positive effect on anxiety level (*p* < 0.01), while married nurses had a slight positive effect on anxiety level (*p* < 0.05). The dependent variable in Table 8 represented the anxiety scale while the predictors included age, education level “high education,” marital status “married,” experience, and job title “bedside nurses.” Furthermore, as stated in Table 9, a highly significant model was detected through the *F*-test value of 15.409 (*p* < 0.01). This model explained 54% of the willingness to report near misses detected through the *R*^2^ value of 0.54. In Table 9, the dependent variable referred to the stress level while the predictors include age, education level “high education,” marital status “married,” experience, and job title “bedside nurses.” Also, the latter model explained that an experience had a high-frequency negative effect on the level of stress (*p* < 0.01). Meanwhile, high education level and age had a slight negative effect on the level of stress (*p* < 0.05). In addition, bedside nurses had a high positive effect on stress level with *p* < 0.01, and the same finding was reported for married nurses.

**Table 6:** Distribution of the studied critical care nurses according to their stress level (*n* = 469).

**Stress domains**	**High**		**Moderate**		**Low**	
	***n***	**%**	***n***	**%**	***n***	**%**
Death and dying patients	161	34.3	210	44.8	98	20.9
Conflict with physicians	133	28.3	189	40.4	147	31.3
Inadequate preparation	168	35.8	224	47.8	77	16.4
Lack of staff support	147	31.3	210	44.8	112	23.9
Conflict with other nurses	98	20.9	189	40.3	182	38.8
Workload	196	41.8	210	44.8	63	13.4
Uncertainty concerning treatment	154	32.8	245	52.3	70	14.9
Total	140	29.8	224	47.8	105	22.4

**Table 7:** Correlations between studied variables.

		**Anxiety level**	**Stress level**	**Coping mechanism**
Anxiety	r.		0.688	−0.619
	p		<0.01^**^	<0.01^**^
Stress	r.	0.688		−0.549
	p	<0.01^**^		<0.01^**^
Coping mechanism	r.	−0.619	−0.549	
	p	<0.01^**^	<0.01^**^	

** *Highly significant*.

**Table 8:** Multiple linear regression (MLR) models for anxiety scale.

	**Unstandardized Coefficients**	**Standardized Coefficients**		
	**B**	**Beta**	**T**	***P* value**
Age	−0.354	0.287	8.011	<0.01^*^
Education level “High education”	−0.299	0.201	4.123	<0.05
Marital status “Married”	0.190	0.135	2.809	<0.05
Experience	−0.344	0.256	6.770	<0.001
Job title “bedside nurses”	0.411	0.367	9.076	<0.01
**Model**	**R** ^**2**^	**F**	***P*** **value**	
**ANOVA**				
Regression	0.52	13.808	*P* < 0.01^**^	

* *Significant*,

** *Highly significant*.

**Table 9:** MLR models for stress level.

	**Unstandardized coefficients**	**Standardized coefficients**		
	**B**	**Beta**	**T**	***P* value**
Age	−0.211	0.134	3.242	<0.05^*^
Education level “High education”	−0.199	0.103	2.998	<0.05^*^
Marital status “Married”	0.305	0.211	7.644	<0.01^**^
Experience	−0.410	0.346	8.066	<0.01^**^
Job title “bedside nurses”	0.398	0.302	7.667	<0.01^**^
**Model**	**R** ^**2**^	**F**	***P*** **value**	
**ANOVA**				
Regression	0.52	13.808	*P* < 0.01^**^	

* *Significant*,

** *Highly significant*.

## Discussion

Health-care workers are exposed to various infectious diseases, including those transmitted through blood or other body fluids and/or airborne infectious agents (12, 24). This study provided interesting baseline information in relation to the psychological stress, anxiety factors, and coping mechanisms of critical care unit nurses during the COVID-19 outbreak in Saudi Arabia. To the best of our knowledge, this is the first study to be conducted on nurses at a national level to explore the psychological stress, anxiety factors, and coping mechanisms of critical care unit nurses.

In accordance with the sociodemographic data and characteristics of the participants, this study showed that more than half of the sample was female, the majority of participants were married, and most of them had children. In addition, nationality was Saudi Arabian for the majority of participants and bachelor's degree constitutes about half of the sample. Nearly, half of the samples were bedside nurses with 6–10 years of working experience in critical care units. This study also reported a median age of 31.73 years. These demographic findings and characteristics of the participants are in agreement with those reported elsewhere (25). The results also emphasized that gender might influence the feeling anxiety and the ability to cope up with stress. In this respect, our results depicted that the total mean score of men was higher than that of women although both have a moderate score. In contrast, a previous study stated that women showed more severe anxiety and fear than men regarding COVID-19 (10). It was documented that women in the nursing society develop various personal and social mechanisms to cope up with stress in comparison with men (26). This discrepancy in the results might be attributed to the possible influence of regional and cultural variations, working environment, and conditions (27, 28). This study also showed that more than half of the participants were very afraid from the contraction of the infection or making other staff or families infected and they were also very stressed about taking care of their infected colleagues and wearing protective clothing for a long time. A possible explanation that COVID-19 outbreaks were severe at the time of this study, and the measures adopted toward disease prevention were not clearly instigated. Similarly, a previous study (10) stated that nurses are among the most vulnerable groups at the core of infection and their worrying about being infected is attributed from close contacts of infected patients, physical discomfort, and facing the death of critically ill patients. Other previous studies (22, 29) revealed that the feeling of stress for critical care unit nurses might result from the awareness of the mortality rate.

Interestingly, this study demonstrated that participants felt nervous, anxious, and afraid from the occurrence of something awful, these findings are in agreement with some previous studies (30, 31). Furthermore, our results revealed that more than one-third and one-quarter of the participants had severe and moderate anxiety levels, respectively. In addition, approximately one-quarter of the participants had mild anxiety. This might indicate the good knowledge and information of some of the participants about the pandemic and reflects the efficacy of different media, including social media, in raising the public health awareness in relation to the distribution of the information about COVID-19 (32). In addition, the factors causing the highest anxiety levels include providing care for infected colleagues and worrying about infecting their family, whereas thinking that the current protection measures are still lacking and constantly screening yourself for infection were the factors causing the lowest anxiety levels. These results are in harmony with the data of Nemati et al. (33) who conducted a study on 85 nurses in Iran and stated that the mean anxiety score was 6.02 ± 2.6 and the score for anxiety about infecting their family was 6.87 ± 2.8. In addition, our present results are consistent with those of Simonetti et al. (34) in which 1,005 nurses employed in different Italian hospital wards had moderate anxiety levels. Similarly, Yanez et al. (35) reported that more than half of the nurses in their study had moderate anxiety levels. Taking into account, several factors such as years of experience, workloads, inadequate preparations, the lack of safe and effective treatment for the disease, the statistics of pandemic and daily reported new cases, and shortages of supplies and equipment, availability of adequate protective measures, and the number of hospitalized cases in critical care units might contribute to the degree of anxiety, fear, and stress during similar pandemics (36, 37).

It is noteworthy to mention that nurses mostly have the highest level of occupational stress among health-care workers as they are often the first frontline health workers who respond to patients (38). More importantly, health-care workers might adjust to a stressful working environment but stressors might have a cumulative effect, resulting in psychological distress. Regarding stress levels, this study revealed that more than one-third and slightly less than half of the participants had high and moderate levels of stress related to their workloads and inadequate preparation, respectively. In accordance with total stress, more than one-quarter and slightly less than half of the participants had high and moderate stress levels, respectively. These findings were in agreement with those of Kar et al. (39) who conducted a study on 733 respondents within 10 days of the survey from 20 countries and stated that only less than one-quarter had stress symptoms. Furthermore, Said and El-Shafei (37) conducted a study on 420 nurses at Zagazig General Hospital and reported that three-quarters of the nurses had high stress levels and most nurses had stress related to workloads. Similarly, Maraqa et al. (40) conducted a study on 430 frontline health-care workers in Palestine and detected that approximately three-quarters reported high stress levels during the outbreak. Fear of transmitting the virus to their family was the most stressful factor, which is consistent with our present findings. It should be stressed that other previous studies reported discrepancies in the results in relation to the level of stress, whereas a wide range of prevalence levels for anxiety and stress (18.1–80.1%) were reported (41). Taking this into consideration, this variation in the level of stress might be attributed to multiple factors that include the possible influences of regional and cultural variations, the level of providing social and moral support, knowledge and preparedness, workloads and inadequate preparations, the lack of proper training and guidelines, and a variation in the methodology in the expression of anxiety and stress (42).

It should be stressed that having proper coping strategies during outbreaks of pandemic remains a critical line in the protection of health-care workers from the contraction of the infection besides their role in the prevention of several stress-related psychiatric disorders (43–45). Clearly, adequate coping strategies together with the social and emotional support are considered as major contributors to the motivation of health-care workers during these pandemic outbreaks. As shown in our present work, critical care nurses use many coping strategies for combating the stress and anxiety caused by the outbreak of COVID-19. The most common coping strategies are displayed in Table 4, which include talking to yourself and motivating yourself for combating the COVID-19 outbreak with a positive attitude, chatting with family and friends to relieve stress, choosing solo transport modes such as self-driving, and avoiding public transportation, doing some leisure activities in your free time, and learning about COVID-19, its prevention, and mechanism of transmission. This study also revealed that more than half, one-quarter, and one-fifth of the participants had low, moderate, and high coping mechanisms, respectively. These results are in agreement with a study conducted by Huang et al. (10) on 804 subjects in China that showed approximately half of the participants had low coping mechanisms. Additionally, our results were consistent with those of Alsolais et al. (46) who detected that most of the participants had moderate coping strategies in Saudi Arabia. However, it should be borne in mind a strong link between individual vulnerability to stress and the used coping strategies during specific situations. Importantly, adequate protective equipment, managemental recognition, and teamwork might reduce the psychological distress of health-care workers during similar pandemics (45). Clearly, public health education of health-care workers about the importance of coping strategies and their effective methods would be very helpful.

Regarding the correlations among the studied variables, which are illustrated in Tables 7–9, this study revealed that age, education level, and years of experience had a high-frequency negative effect on the level of anxiety and stress, reflecting the possible influence of age and years of experience of participants on the reduction of the level of anxiety and stress. However, previous reports revealed that all age groups of health-care workers expressed psychological stress when they saw their colleagues under stress (45, 47). In stark contrast a previous study (48) in Jordan documented that older health-care workers had a higher level of psychological distress that might be related to a higher risk of severe multiple organ and respiratory failure among elderly during COVID-19 outbreaks. In the same study (48), a weak correlation was reported between years of experience and fear and anxiety, which could be attributed to the uncertainty of health-care professional safety, the regular reuse of personal protective equipment potentiated, and attending severe complicated and death cases (49). Furthermore, there was a strong positive correlation between anxiety and stress levels (*p* < 0.01). Meanwhile, there was a strong negative correlation between coping mechanisms and stress and anxiety levels (*p* < 0.01). These results are similar to those reported by Lorente et al. (50) on 421 nurses from 39 Spanish provinces. This study showed that emotion-focused strategies were negatively related to nurses' psychological distress directly and indirectly through resilience. Similarly, Lou et al. (51) studied 115 subjects in Montreal, Canada, and reported that adaptive coping strategies moderated a negative impact of stress on work performance and also a negative effect of stress on burnout. Additionally, our findings are in harmony with those of Vagni et al. (52) who conducted on 121 nurses in Italy and revealed that coping mechanisms caused to decrease anxiety levels in nurses. In addition, this study reported that bedside nurses had a positive effect on anxiety and the stress level that reflects the more anxiety and the stress level could be found among bedside nurses. A possible explanation for this finding is the close proximity of bedside nurses with critically ill patients, and they usually spend more time and energy to be in close contact with the patients in critical care units. Furthermore, bedside nurses are always struggling to manage and coordinate their professional duties with their own life and family members, making them feel uncertain and unprotected and as a consequence increasing the level of anxiety and depression among these nurses (45). Moreover, the majority of bedside nurses are young health-care professionals, which make them afraid of being infected and died besides their fear from infecting other members in their families. Taking this into account, bedside nurses experienced a sharply deteriorating stage of the disease, which further increase fear and anxiety from being infected (53). In addition, the present findings revealed that the marital status of the study participants could be positive predictors for exploring the level of stress and anxiety that means the level of stress and anxiety is higher among married nurses. Similarly, a recent study documented that 44.4% of married nurses who have children and 29.4% of the nurses working in critical units experienced a high stress (54). Another study revealed that having children and stigmatization are among the relevant factors related to health-care workers' stress (55). A possible explanation of this finding that nurses are always worried about the health of their family as a result of the infection by COVID-19 (56). Collectively, the studied variables reveal that nurses exhibiting high levels of stress, anxiety, and fear from the contraction of the infection do not enact proper coping approaches, and consequently they might have a higher risk and vulnerability. Clearly, proper adaptive coping strategies and approaches are recommended for health-care workers to minimize the degree of stress, arousal, and possibly secondary trauma, which might require special attention.

The limitations of this work, including a limited number of hospitals, the number of participants for the pilot study, a self-report study, and the findings, may be somewhat dated. Furthermore, data were collected through an online electronic questionnaire, which might hinder an accurate observation of nurses' reactions toward stress regarding COVID-19 and read verbal and nonverbal reactions of coping. Similarly, this study was focused on critical care unit nurses, and extending this study to include nurses among emergency departments would be interesting.

## Conclusions and Recommendations

This study concluded that more than one-third and one-quarter of the participants had severe and moderate anxiety levels, respectively. In addition, the highest factors causing anxiety were providing care for infected colleagues and worrying about infecting their family. Moreover, more than one-quarter and slightly less than half of the participants had high and moderate stress levels, respectively. More than half of the studied nurses had low coping mechanisms, and one-quarter of them had moderate coping mechanisms. There was a strong positive correlation between anxiety and stress levels and also between coping mechanisms and stress and anxiety levels. The main factors associated with stress in this study included the perceived risk of infection to themselves and their families, the care of infected colleagues, and wearing protective clothing for a long time.

This study recommends continuous educational programs for nurses on coping mechanisms, which should be adopted together with the framing of preventive measures and a psychological intervention plan within a mandatory occupational health surveillance program. These measures should be supported by policymakers to protect frontline health-care workers during disease outbreaks. Also, nurses should develop personal coping strategies through constant education; regular vacations from their work and psychological stress should be minimized to fulfill high-quality nursing care, aiming at the prevention and reduction of fear and anxiety, and stress. Further research is suggested with a larger sample size, and it would be also interesting to evaluate whether changes occur over time. In addition, this study should be applied to all hospitals in the Kingdom of Saudi Arabia to explore more about stresses and more mechanisms of coping and adaptation. Future research is also suggested about specific stressors and their pathogenesis on health-care workers to be able to develop individual stressor management or the possible treatment of stress.

## Data Availability Statement

The original contributions presented in the study are included in the article/supplementary material, further inquiries can be directed to the corresponding author/s.

## Ethics Statement

The studies involving human participants were reviewed and approved by the Research Ethics Committee of Jouf University and the Study Approval No 05-06-42. The submission of the answer to the questionnaire was considered as consent to take part in the study. Confidentiality of the study subjects' data was sustained throughout the study by making the mothers' data nameless. The patients/participants provided their written informed consent to participate in this study.

## Author Contributions

SA and SD contributed to the conception and design of this study, data collection, analysis, interpretation, manuscript writing, and reviewing and revising the manuscript. EE contributed scientific advice and prepared the manuscript for publication and revision. All authors read and approved the final manuscript.

## Funding

This work received research Grant No. (DSR2020-04-2545) from Jouf University.

## Conflict of Interest

The authors declare that the research was conducted in the absence of any commercial or financial relationships that could be construed as a potential conflict of interest.

## Publisher's Note

All claims expressed in this article are solely those of the authors and do not necessarily represent those of their affiliated organizations, or those of the publisher, the editors and the reviewers. Any product that may be evaluated in this article, or claim that may be made by its manufacturer, is not guaranteed or endorsed by the publisher.
